# A Computational Approach towards the Understanding of *Plasmodium falciparum* Multidrug Resistance Protein 1

**DOI:** 10.1155/2013/437168

**Published:** 2013-08-01

**Authors:** Saumya K. Patel, Linz-Buoy George, Sivakumar Prasanth Kumar, Hyacinth N. Highland, Yogesh T. Jasrai, Himanshu A. Pandya, Ketaki R. Desai

**Affiliations:** ^1^Department of Bioinformatics, Applied Botany Centre (ABC), University School of Sciences, Gujarat University, Ahmedabad 380009, India; ^2^Department of Zoology, Biomedical Technology and Human Genetics, University School of Sciences, Gujarat University, Ahmedabad 380009, India

## Abstract

The emergence of drug resistance in *Plasmodium falciparum* tremendously affected the chemotherapy worldwide while the intense distribution of chloroquine-resistant strains in most of the endemic areas added more complications in the treatment of malaria. The situation has even worsened by the lack of molecular mechanism to understand the resistance conferred by Plasmodia species. Recent studies have suggested the association of antimalarial resistance with *P. falciparum* multidrug resistance protein 1 (PfMDR1), an ATP-binding cassette (ABC) transporter and a homologue of human P-glycoprotein 1 (P-gp1). The present study deals about the development of PfMDR1 computational model and the model of substrate transport across PfMDR1 with insights derived from conformations relative to inward- and outward-facing topologies that switch on/off the transportation system. Comparison of ATP docked positions and its structural motif binding properties were found to be similar among other ATPases, and thereby contributes to NBD domains dimerization, a unique structural agreement noticed in *Mus musculus* Pgp and *Escherichia coli* MDR transporter homolog (MsbA). The interaction of leading antimalarials and phytochemicals within the active pocket of both wild-type and mutant-type PfMDR1 demonstrated the mode of binding and provided insights of less binding affinity thereby contributing to parasite's resistance mechanism.

## 1. Introduction

The failure of commonly used antimalarial agents in treating chloroquine-resistant *Plasmodium falciparum* had complicated the management of malaria in most of the developing countries including India. WHO reported an estimation of 216 million malaria cases worldwide in 2010 and around 1.5 million confirmed cases annually with 50% accounting for *P. falciparum* resistance according to the National Vector Borne Disease Control Programme (NVBDCP), India, 2011 [1, 2]. Recent studies have showed a strong association between chloroquine-resistant strains and the molecular changes in *P. falciparum* multidrug resistance protein 1 (PfMDR1) [3, 4]. PfMDR1 is a member of the ATP-binding cassette (ABC) superfamily and a transporter protein involved in small molecule trafficking. The functional polymorphisms encoded by *pfmdr1* gene mutations lead to the development of resistance against leading antimalarial agents including chloroquine [5]. 

PfMDR1, a transmembrane glycoprotein and a homologue of P-glycoprotein 1 (P-gp1), is classified under the protein superfamily, ABC transporters, which act as efflux pumps that help in substrate translocation including the antimalarial agents and have been linked to multidrug resistance in malaria [6] and cancer [7]. Notable single nucleotide polymorphism in the *pfmdr1* gene modulates drug susceptibility, and the physiological mechanism at the protein level is still unknown [8]. The present study aims to understand the molecular mechanism of PfMDR1 substrate transportation cycle wherein the structural details and interaction of ATP and antimalarials as well as the model for substrate transport are elucidated. Ferreira and coworkers, 2011, developed a homology model of PfMDR1 protein by considering *Escherichia coli *MsbA (open and apo conformation; PDB entry: 3b5w; identities: 26% and similarities: 47%), *Vibrio cholerae* MsbA (closed and apo conformation; 3b5x; 28% and 46%), and *Salmonella typhimurium* MsbA (open and nucleotide-bound conformation; 3b60; 26% and 46%) as templates and studied its interaction with antimalarials [9]. We selected domain specific templates which differ from templates chosen by Ferreira et al., 2011 to study the proposed PfMDR1 protein model. 

## 2. Materials and Methods

### 2.1. Sequence Analysis and Multiple Sequence Alignment of Target with Template Proteins

The primary sequence of PfMDR1 was retrieved from UniProtKB database [10] with the accession number P13568. Blastp [11] homology search over RCSB PDB database [12] using expect threshold of 10 and BLOSUM62 scoring matrix [13] identified structural templates. Based on the query coverage, identities, and E (expected) value, the crystal structures of PfMDR1 homologues, namely, *Mus musculus* P-gp1 (PDB entry: 3g5u chain B) [14], *Bacillus stearothermophilus* UvrA endonuclease (2r6f chain A) [15], and *Saccharomyces cerevisiae* elongation factor (eEF3; 2iw3 chain B) [16] were obtained from PDB and subjected to multiple sequence alignment using EBI ClustalW program [17] to sort out the identical domains and insertions/deletions (INDELs). PfMDR1 protein profiles and patterns were studied using ExPASy PROSITE database with ScanProsite tool [18]. The hydrophobicity of PfMDR1 was estimated using ProtScale program [19] with Kyte-Doolittle amino acid scale [20].

### 2.2. Construction of the Homology Model and Energy Minimization

The homology model of PfMDR1 was constructed using structural homologues using Modeller v9.10 program [21] that implements comparative protein structure modeling by satisfaction of spatial restraints. The loops were refined by an automated optimization process based on conjugate gradients approach and molecular dynamics with simulated annealing method. The generated models were selected by examining their internal scoring function, DOPE (discrete optimized potential energy) score. The structural arrangement with respect to template structures was graphically visualized using UCSF Chimera program [22] by superimposition. Further, energy minimization of protein models was carried out using KoBa^MIN^ webserver [23] to fix the protein side chains and to obtain a realistic conformation. This program refines protein structure by utilizing a statistical knowledge-based potential method initially followed by stereochemistry correction using MESHI program [24]. The KB01 energy was estimated using ENCAD force field [25] and KB01 potential terms, whereas MESHI stage utilizes standard energy parameters. The global distance test (GDT_TS) for average structures and high accuracy (GDT_HA) score at 1 Å cutoff was inspected to understand the quality of minimized model with respect to its native structure.

### 2.3. Assessment of the Homology Model and Structural Superpositions

The quality of the selected PfMDR1 model was assessed by Ramachandran plot [26] statistics which is based on the distribution of phi (Φ) and psi (*ψ*) torsion angles of backbone protein conformation, implemented in VADAR (Volume Area Dihedral Angle Reporter) web server [27]. The structure reliability was checked by profile quality index using 3D profile assessment, a unique feature of VADAR. Finally, the probable protein folding energy of the theoretical model was studied by ProSa II program [28] which compares energy criteria with the potential mean force derived from a large set of experimental protein structures. Native (model without energy minimization) and minimized conformers of PfMDR1 and templates were structurally superposed using FATCAT [29] and CLICK [30] programs, respectively. FATCAT (Flexible structure AlignmenT by Chaining Aligned fragment pairs allowing Twists) aligns protein structure flexibly by optimizing and minimizing the number of rigid-body motions (twists) around pivot points (hinges) observed in the protein native structure [29]. CLICK algorithm depends on matching cliques of points within a certain spatial proximity defined by the pairwise distance threshold, and the points were weighted by structural features including secondary structure, solvent accessibility, and depth [30].

### 2.4. Prediction of Interface Site and Protein-Protein Docking

The interfacial amino acids participating in the protein-protein interaction were enumerated using WHISCY [31] and PredUs [32] programs, respectively. Both of these programs utilize structural conservativeness as the main stream for prediction wherein WHISCY considers surface smoothing and accessibility, whereas PredUs uses support vector machine (SVM) to distinguish interfacial from noninterfacial residues using the knowledge of structural representatives clustered by CD-HIT program [33]. PfMDR1 structural units undergo dimerization which transforms inward-facing conformation to outward. The molecular motions of PfMDR1 from inward to outward topology were studied by protein-protein docking simulations using Escher NG docking system [34] of VegaZZ program (academic license) [35]. The entire generated frames were considered and sorted by energy threshold. The best docked conformer was selected based on the docking score and the structural resemblance to template in its outward-facing conformation. Evaluation of surface complementarity, hydrogen bonding, and electrostatic interaction in molecular recognition—next generation (ESCHER NG) is a docking procedure that employs geometric complementarity method to generate a set of rough solutions followed by refinement of atomic collisions and finally evaluated by electrostatic complementarity [34].

### 2.5. Ligand Dataset Retrieval and Small Molecular Docking

The structures of selected antimalarials (amodiaquine, artemisinin, calotropegenin, chloroquine, halofantrine, lumefantrine, mefloquine, vinblastine, and vincristine) were downloaded from NCBI PubChem database [36] and subjected to energy minimization using YASARA Structure [37] based on Amber03 force field [38] to obtain a stable conformation. Molecular docking simulations within the PfMDR1 drug-binding pocket were performed using AutoDock 4.2 [39] operated in Windows 7 Ultimate environment with 4 GB RAM and Intel Core i5 processors. The protein structure file was preprocessed by removing water molecules, adding polar hydrogens, and assignment of Kollman charges [40] using AutoDock Tools 4 (ADT4). Gasteiger partial charges [41] were assigned and the torsional restraints were made flexible in order to obtain favorable binding conformation. AutoGrid 4.2 [39] was used to generate grid maps centered on the drug-binding pocket with the following settings: number of points in dimensions = 80 × 100 × 58 with points separated by 0.375 Å, grid dimensions = 13.457 × 42.632 × 40.03 Å^3^. Standard docking protocol was considered which returned 100 GA (genetic algorithm) runs per ligand with an initial population of 250 randomly placed individuals, maximum number of energy evaluations limited to 2.5 × 10^5^, and rate of gene mutation and crossover constrained to 0.02 and 0.8. The probability of observing a local search on each individual was expected to be 0.06 with a maximum of 1000 iterations per search. The docking simulations resulted in 100 solutions which were clustered by root mean square (RMS) deviation and binding energy in which a lowest energy conformer was selected to understand the receptor-ligand interaction patterns.

## 3. Results and Discussion

### 3.1. Structural Features of PfMDR1

PfMDR1, a homologue of human P-gp1 and a member of the highly conserved superfamily of ATP-binding cassette (ABC) transporter protein, acts as an energy-dependent efflux pump that facilitates transporting structurally diverse small molecules and has been implicated in multidrug resistance of the parasite. PfMDR1 consists of two symmetric “halves” spanning ~130.60 Å perpendicular and ~90 Å in the membrane bilayer plane and modeled as nucleotide-free inward-facing conformation having a resemblance to inverted V-shape, a characteristic apo form of bacterial ABC lipid flippers [42].

**Figure 1:**
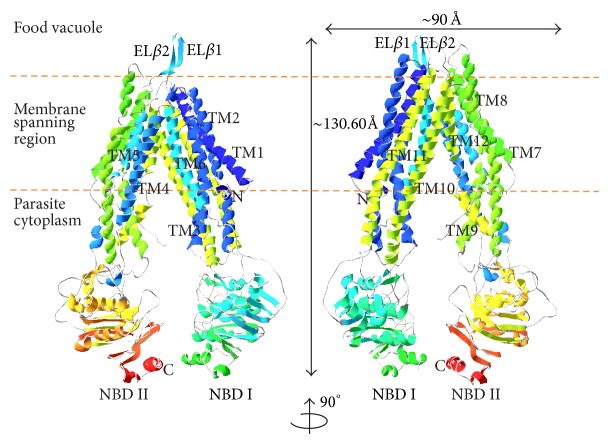
3D model of developed PfMDR1.

PfMDR1 can be distinguished into two domains, designated as domains I and II according to its terminal location, N and C. Each domain can further be divided into transmembrane domain (TMD; TMD I residues: 56–338 and TMD II residues: 789–1083) and nucleotide-binding domain (NBD; NBD I residues: 339–788 and NBD II residues: 1084–1419). The TMDs are encompassed with transmembrane (TM) spanning helical bundle which is organized into three external loops (EL) and two internal helices (IH) that colligate six TM helices (Figure 1). The NBDs possess prominent structural features essential for basal ATPase activity and bear more similarity to *M. musculus* P-gp1 (PDB entry: 3g5u chain B, identities: 28.9%, similarities: 46.3%). PfMDR1 NBDs also share similarity with UvrA endonuclease of *B. stearothermophilus *(2r6f chain A, 17.7%, 32.5%) and *S. cerevisiae* elongation factor (eEF3; 2iw3 chain B, 12%, 20.9%) (Supp. Figure 1 in Sypplementary Material available online at http://dx.doi.org/10.1155/2013/437168). It should be noted that the above percentage of identity and similarity were ascribed to the alignment of the PfMDR1 NBDs domain with respective structural homologs. In addition, the N-terminal region harbors 55-amino-acid long topological domain. Homology search over NCBI nonredundant database identified the existence of this topological domain (residues: 1–55) in other strains of *Plasmodium* including *P. knowlesi* strain H (identities: 60%, similarities: 75%), *P. vivax* Sal-1 (60%, 77%), *P. berghei* strain ANKA (65%, 82%), *P. chabaudi* (57%, 80%), and *P. yoelii yoelii* (65%, 80%). This domain also exhibited a disorderness profile with confidence intervals of 8 and 9. 

The PfMDR1 computational model was developed based on the structural homologs of ABC transporter proteins. A Blastp search over the PDB database yielded six structural templates with more than 87% comprehensive sequence coverage *E* value in the range of 2*e* − 56 to 6*e* − 176. Fortunately, all the blast hits were from ABC members which significantly boosted up the confidence of obtaining a reliable model. Since PfMDR1 is a membrane-bound protein, we carefully examined the regions of TM and those of NBDs having similarity to plausible templates. The TMs spatial organization mimicked the typical ABC membrane transporters, whereas NBDs crucial for ATPase activity shared structural motifs of ATP binding and catalysis. *M. musculus* P-gp1 scored overall 29% identities, 48% similarities, and 16% gaps when aligned with PfMDR1 primary sequence. The gaps produced were attributed to the compositionally biased poly-Asn region at the sequence span of 643–661 and the ELs in the respective protein structures. These gaps are due to INDELs in ELs that are functionally annotated as requisite structural elements for convenient TMs interlocking and to enhance interchain contacts as observed in *Escherichia coli* MDR transporter homolog (Eco-msbA) [43]. 

Intertwining TMs form the major channel for transporting small molecules and interconnect NBDs via IHs. The principal sequence variation was largely observed on ELs, namely, EL1-3, whereas the rest of the ELs persist as conserved. Sequence insertion was noticed on the regions of EL1, EL*β*1-2, loops connecting *α*4 and *α*5, *β*9 and *α*11 and TM10. NBD I comprises 10 *α*-helices and 9 *β* strands while NBD II contains 10 *α*-helices and 11 *β* strands. Interlocking of TM1-3, TM4-6, TM7-9, and TM10-12 builds up two hinges which were subjected to molecular motions during ligand movement across the channel as observed in Eco-msbA. Further, structural details of Eco-MsbA indicated the preference of EL1 and EL6 towards covalent interaction by establishing spontaneous disulphide bonding in ATP-unbound form (apo form) [43]. Hence, we compared the structural arrangement of EL1 and EL6 in the modeled PfMDR1 with respect to Eco-MsbA which revealed that residues making up the loop elements are observed in close proximity. Amino acids such as Asn84, Met85, Asn86 and Leu87 of EL1 and Phe1051, Leu1052, Ile1053, Lys1054, Arg1055, Gly1056, Lys1057, Ile1058, Leu1059, and Val1060 of EL6 are involved in this interaction. Each NBD (NBD I and II) can further be subdivided into ATP-binding domain (ATP-binding domain I residues: 339–562 and 578–788, ATP-binding domain II residues: 1084–1311 and 1327–1419) and ATPase signature domain (ATPase signature domain I residues: 563–577 and ATPase signature domain II residues: 1312–1326).

PROSITE profile search over the UniProtKB database recognized two distinct profiles, ABC transporter integral membrane type-1 fused domain profile (residues: 58–345 and 791–1083; score: 33.159 and 32.343) and ABC-binding cassette (residues: 378–662 and 1126–1416; score: 22.149 and 19.961). Pattern hits predicted a unique family signature, ABC transporters family signature in the sequence positions of 563–577 (sequence: LSSGGQKQRISIARAI) and 1312–1326 (sequence: LSGGQKQRIAIARAL) and corresponded to ATPase signature domain I and II, respectively (Suppl. Figure 2). 

### 3.2. Structure Verifications

The hydropathy plot delineated by Kyte-Doolittle scale [20] boosted up the prediction of TMD I and II with a score in the range of 2.3 to 3.1 at a threshold of 1.8 (Suppl. Figure 2). Energy minimized structures of P-gp1 (template) and PfMDR1 (model) were retrieved from KoBa^MIN^ server [23]. The KB01 energies for P-gp1 and PfMDR1 were found to be −30129.97 kcal/mol and −21478.62 kcal/mol, respectively. The metrics employed for structural assessment gave a meaningful comparison of the template and the model. The GDT_TS scores for the template and developed model were reported to be 0.932 and 0.933 at 1 Å cutoff, whereas the GDT_HA scores (P-gp1: 0.757 and PfMDR1: 0.760) were examined at 0.5 Å for improved accuracy which indicated that the model developed has a good agreement with the template. Subsequently, stereochemistry checking was also carried out to understand the structural packing. Ramachandran plot [26] revealed a similar empirical distribution of amino acid datapoints in contrast to template over Φ/*ψ* space wherein the majority of amino acids was disseminated in favored regions (P-gp1: 61.9%, PfMDR1: 62.9%); some residues attributed to TM regions were dispersed over generally allowed areas (P-gp1: 26.8%, PfMDR1: 25.9%) and few outliers (P-gp1: 11.3%, PfMDR1: 11.2%). It can be noted that the amino acid distributions of developed model plotted over various regions followed a similar trend with respect to template plot. The quality of the constructed protein model was also studied by 3D profile quality index to assess the local environment and packaging.

**Figure 2:**
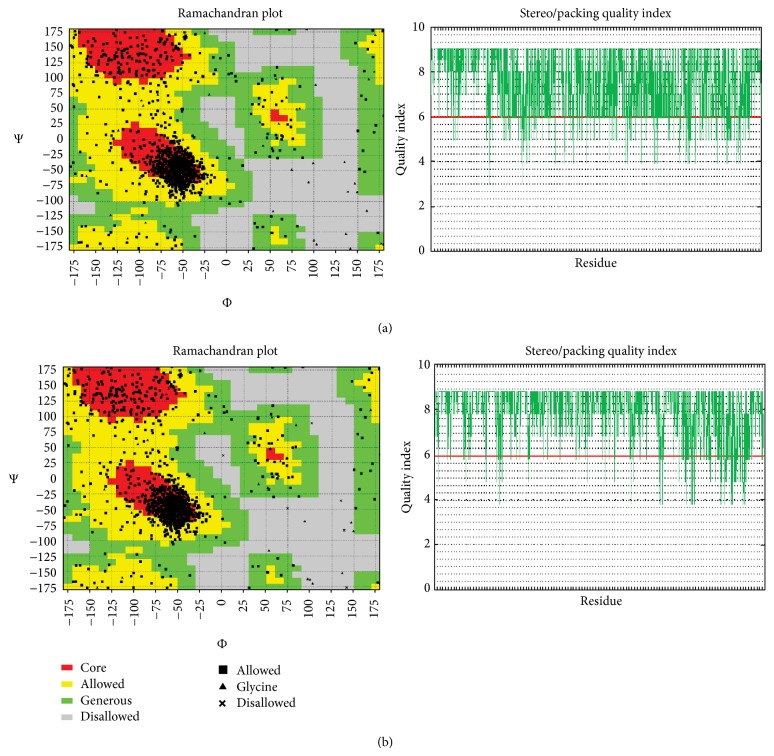
Ramachandran map and quality index of template *M. musculus* P-gp1 (a) and modeled PfMDR1 (b).

The profile of the PfMDR1 model resembles its template protein, P-gp1, and the majority of the protein residues were assigned with a high confidence intervals of 5 and 8 suggesting the reliability of the local structure with exceptions over NBD II quality data points in both structures (Figure 2). 

In addition, ProSA provided an energy-based qualifier, *Z*-score, to recognize structural errors. The *Z*-scores for P-gp1 (−12.63) and PfMDR1 (−9.29) were found to be plausible and this overall negative score was carved up by negative scoring energy characteristic of TM regions and the positive energy by globular form of NBD I and II. The role of this indicator on membrane spanning regions was perplexed due to the derivation of structure quality indices from soluble proteins and their role in membrane and its associated proteins remains ambivalent (Suppl. Figure 3). However, *Z*-score can be used as a diagnostic tool to understand the level of score deviations provided a particular protein class has been considered. Hence, it is evident from the close values of template and theoretical model which showed the regularity of protein structures. 

**Figure 3:**
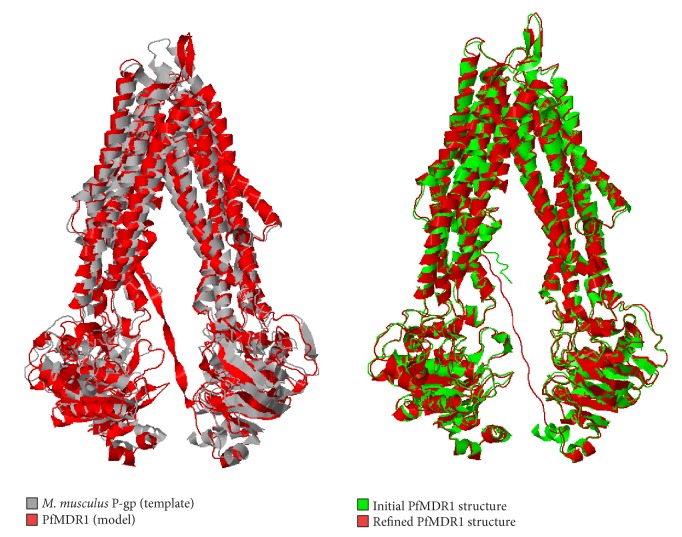
Structural superposition of *M. Musculus P-gp* and PfMDR1 model.

Structural superimposition of energy minimized conformers of P-gp1 and PfMDR1 resulted in structural alignment with RMSD of 3.10 Å over 1134 equivalent positions. The conformational flexibility of PfMDR1 was optimized by implementing FATCAT program which considers minimization of rigid-body movements including twists and hinges with an RMSD of 0.97 Å and aligned over 1154 matched positions with respect to PfMDR1 model without minimization (Figure 3). Loops interlinking TMs, NBDs, and ELs were subjected to substantial refinement and the refined PfMDR1 model was employed in further studies. 

### 3.3. Model of Substrate Transport by PfMDR1

The inward-facing conformation represents open apo form (drug-unbound) of PfMDR1 with inverted V-shape topology, whereas the closed drug-bound conformation is associated with outward-facing structural configuration with V-shaped topology. The PfMDR1 was modeled based on the P-gp1 protein structure in its inward-facing conformation which is known to be the representative structure competent for drug binding in its initial stage of substrate transportation cycle. The drug-bound conformation of P-gp1 is due to the substrate-stimulated ATPase activity on NBDs (NBD I and II) which results in NBDs dimerization [13]. This outward-facing conformation is also observed in MsbA [43] and Sav1866 [44] wherein the NBDs interaction supports the model of substrate transport. It should also be noted that this dimerization facilitates substrate export to food vacuole thereby inhibiting substrate translocation towards parasite cytoplasm. 

**Figure 4:**
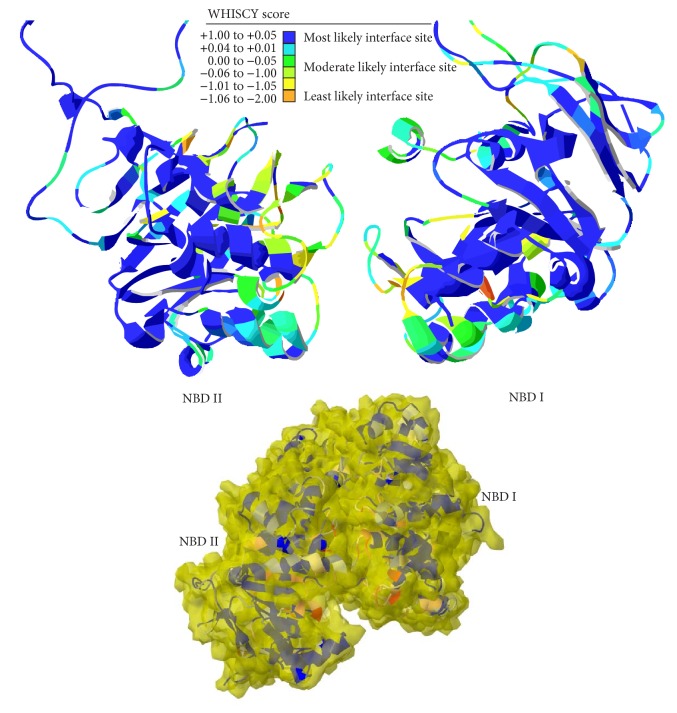
WHISCY predictions at the NBD dimer site of PfMDR1.

To develop a dimeric state of PfMDR1 model, we examined the structural preference of interacting sites at the NBD domains using WHISCY prediction program [31]. WHISCY predictions are based on structural conservativeness precompiled by protein sequence homology and combines structural information to cipher surface smoothing. The surface smoothing is subsequently refined by WHISCYMATE program utilizing the ability of ProMate [45] to compute interface propensities by probing surface dots at 10 Å radius circle. This calculation returned prediction scores along with a customized PDB file. Blastp suggested PfMDR1 homologues at a threshold value of 10 to generate multiple sequence alignment file in Clustal format, and a set of surface dots were enumerated using PfMDR1 PDB file at a constant density to predict the interface propensities. The WHISCY score helped us to distinguish the most likely interface sites amongst various classes of predictions. The most likely interface sites (scored with a range of 0.05 to 1.00) were attributed to the surface elements whose accessible surface area (ASA) was found to be greater as expected. It is noteworthy to speculate that the most likely interface predicted by WHISCY on both of the NBD domains is at least stereographical in view indicating the structural interface unit for NBDs dimerization (Figure 4). 

The WHISCY predictions were cross-validated by utilizing PredUs program which also works on the structural conservative background and relies on SVM model trained using structural neighbors clustered by CD-HIT at 40% sequence identity cutoff. On examining the interfacial score, we sorted out the residues preferred to engage in NBDs dimerization. As anticipated, the interface sites on NBD I and II were dominated by polar amino acids with few charged ones (NBD I: 33 residues; NBD II: 26 residues). The frequency of neutral residues was greater including Ser, Asn, Gln, and Thr on both of the NBD domains. Charged amino acids such as Arg, Lys, Asp, and Glu were also observed but to a lesser extent giving clues over the preference of structural complementary regions for NBDs dimerization rather than electrostatic interaction (Table 1).

**Figure 5:**
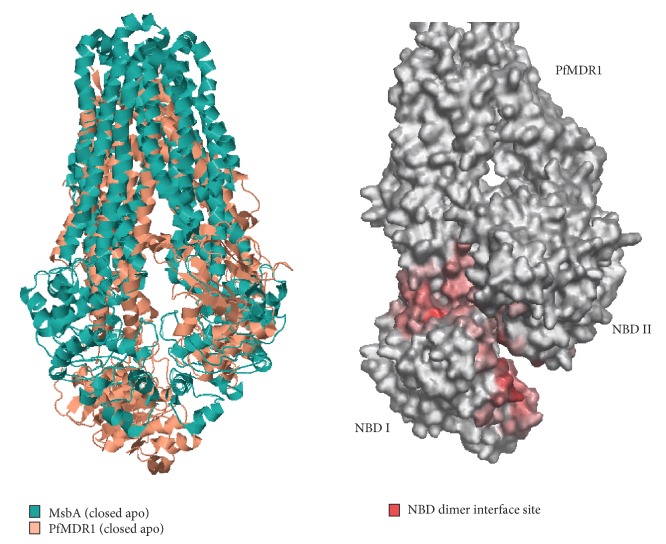
Structural superposition of Eco-MsbA and PfMDR1 in their closed-apo forms (the NBD dimer sites of PfMDR1 are highlighted in red patches).

In order to construct an outward-facing conformation of PfMDR1 model, we relied on the structural details of MsbA in its closed-apo form (ATP unbound in NBD domains) [43] which exemplify the structural transformations carried out for substrate export. Since we focused on capturing these transformations, the protein-protein docking simulations were considered in which the PfMDR1 protein chains A and B were specified as inputs using Escher NG automated docking system implemented in VegaZZ [35] project. Docking runs resulted in 1000 frames which were subsequently clustered based on interaction energy, and the best frames were evaluated. The best cluster was recognized by docking score falling within a range of 821.60 to 900 with 14 candidates, wherein the conformer scoring 880 was chosen due to its close resemblance to MsbA template. The PfMDR1 docked conformer also represented NBDs dimerization with key amino acids predicted previously facilitating interaction. An RMS of 40.4 Å and bumps of 1112 showed that the PfMDR1 was subjected to reasonable large conformational changes (Figure 5).

The outward-facing conformation was studied by superimposing the docked conformer of PfMDR1 (closed-apo form) with Eco-msbA (closed-apo form) using CLICK [30] program. We selected Eco-MsbA as reference structure and aligned PfMDR1 structure without twists and clique detection method. This method can identify pairwise distances constrained by secondary structures and solvent accessibilities to recognize cliques followed by global alignment of matched cliques. This topology-independent comparison method helped us to identify the best superposed form of PfMDR1 (RMSD: 2.57 Å; match size: 217) with respect to Eco-MsbA (Figure 5). Hence, it is anticipated that the closed form of PfMDR1 might open up its portals due to large conformational change. Consequently, the binding affinity of substrates might get decreased due to alteration in amino acid contacts or due to a mutation event in active site leading to substrate export towards the outer leaflet, that is, extracellular space of food vacuole. In addition, ATP hydrolysis on NBDs causes interference in dimerization and resetting the transport system to inward-facing conformation [46].

 A covalent interaction through spontaneous disulphide bonding by cross-linking experiment was established between EL1 and EL6 making it proximal to each other, which has been known to be one among the prominent characteristic features observed in ATP-unbound form of Eco-MsbA [47]. Similar structural arrangement was also noticed in *M. musculus* P-gp1 [14] and examined in the developed closed-apo form of PfMDR1 which revealed, EL1 and EL6 are very close to each other thereby bringing together the leaflet endings of TM1 and TM11 (Suppl. Figure 4). The closeness of EL1 and EL6 resembled the shape of inverted V shape and formed the hinge of the PfMDR1. It can be demonstrated that the flexibility of these external loops can guide switching the inward and outward conformations and, henceforth, fluctuate substrate transportation.

### 3.4. ATP Interaction at NBD Domains

**Figure 6:**
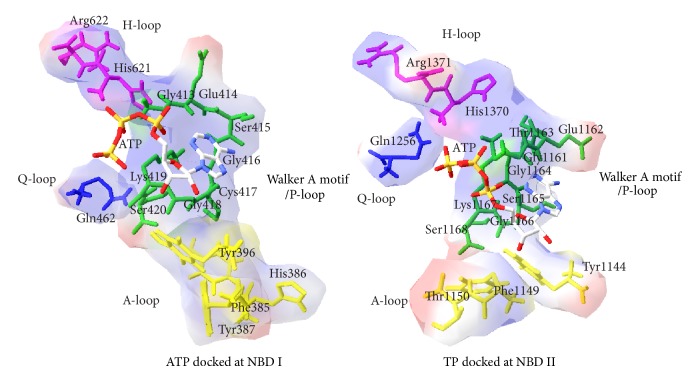
ATP docked view of PfMDR1 NBD I and II.

PfMDR1 possesses tandem ABC ATPase constituting nucleotide-binding sites on NBD I and II which can further be distinguished into ATP-binding domain and ATPase signature domain. Each ATP-binding domain possesses Walker A motif, Q-loop, and H-loop while the ABC signature domain comprises ABC signature motif and D-loop. This structural arrangement bears more resemblance to *B. stearothermophilus *UvrA endonuclease nucleotide-binding sites [15]. Walker A motif is otherwise known as P-loop (phosphate-binding loop) with GXXXXGK(T/S) pattern, where G, K, T, S, and X are glycine, lysine, threonine, serine, and any of the 20 natural amino acids, respectively. This consensus sequence pattern was observed in the NBDs I and II in the span of 413–420 (GESGCGKS) and 1161–1168 (GETGSGKS), respectively. This motif binds the *β*- and *γ*-phosphates of ATP. The A-loop (A stands for aromatic residue) comprises a highly conserved aromatic amino acid which is spaced 25 residues upstream to Walker A motif known to be essential for ATP binding as revealed in site-directed mutagenesis experiment [48]. This loop interacts with adenine ring of ATP, wherein Phe385, His386, Tyr387, and Tyr396 of NBD I and Tyr1144, Phe1149, and Thr1150 of NBD II were in close proximity to the adenine ring of ATP docked poses in both NBD domains. Gln462 of NBD I and Gln1256 of NBD II form the core element of Q-loop and function as interconnector of ATP-signature domains with ATP-binding domains which have been proposed to be the site of conformational changes in order to couple ATP hydrolysis [49]. The H loop is required for ATP hydrolysis activity, whereas the binding affinity for ATP remains preserved in *M. musculus* P-gp1 [50]. PfMDR1 contains histidine residues in the positions of 621 of NBD I and 1370 of NBD II and is found to be an upstream element to ATP-signature domain (Figure 6).

 A single key mutation, Asp1246Tyr, an amino acid of *α*13 and 9th amino acid upstream to Q-loop of NBD II, was strongly associated with the alteration in PfMDR1 kinetics only when coupled by mutations in TMDs, namely, Ser1034Cys and Asn1042Asp, respectively [5]. Besides, the Asp1246Tyr mutation exhibits the most basal ATPase activity [5].

### 3.5. Drugs Interaction with PfMDR1

**Figure 7:**
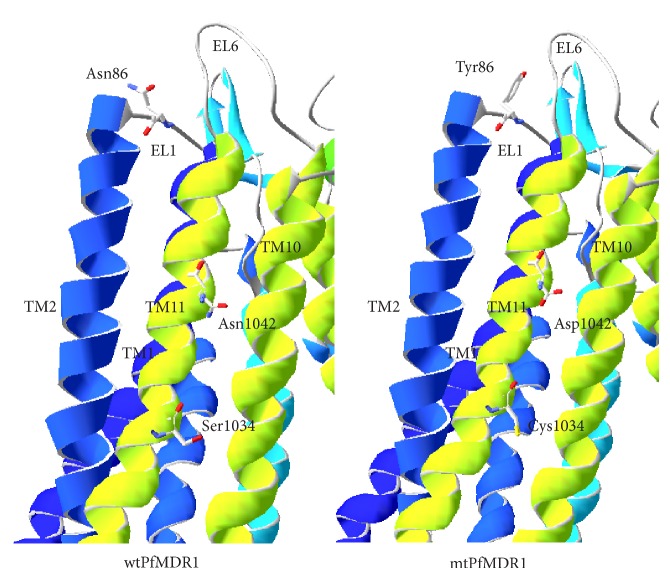
Structural view of SNP in wild-type (left) and mutant-type PfMDR1 (right).

The TMD regions in PfMDR1 form the drug-binding pocket for efficient transportation in which the following amino acids play a vital role *in vitro*: Asn86, Ser1034, and Asn1042 (Figure 7). This drug-binding pocket localizes very close to the outer leaflet of TMDs facing towards the food vacuole in which Asn86 of EL1, Ser 1034, and Asn1042 of TM11 (wild-type) form the core element of drug-binding pocket and their localization is very convenient as PfMDR1 in its outward-facing conformation open up its portals for drug exit. Investigation of antimalarial resistance using *pfmdr1* allelic exchange experiments revealed another functional amino acid, Tyr184Phe of TM3, which appears to interfere in kinetics without affecting the drug specificity [5]. Hence, this mutation was not considered for the present study toward the interaction of drugs in binding pocket. The decreased binding affinity of drugs was attributed to the mutational form of key amino acids including Asn86Tyr, Ser1034Cys, and Asn1042Asp, respectively. Henceforth, we investigated the binding mode of antimalarials in wild-type (wtPfMDR1) as well as mutant type (mtPfMDR1) (Table 2). To develop mtPfMDR1, the reported functional amino acids were subjected to *in silico* mutagenesis and refined through energy minimization protocol discussed earlier.

**Figure 8:**
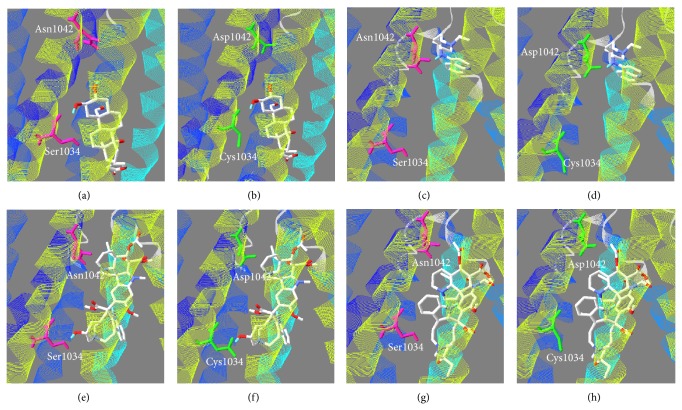
Docked poses of (a-b) calotropegenin, (c-d) chloroquine, (e-f) vinblastine, and (g-h) vincristine in wtPfMDR1 and mtPfMDR1.

Differential *in vivo* and *in vitro *parasite responses to wt- and mt-PfMDR1 against antimalarials including amodiaquine, artemisinin, chloroquine, halofantrine, lumefantrine, mefloquine, vinblastine, and vincristine was reported in the literature [51–56]. The binding mode of these antimalarials was investigated using molecular docking technique (Figure 8). 4-Aminoquinoline drugs such as amodiaquine and chloroquine are especially known for their implications in treating erythrocytic plasmodial infections. Amodiaquine is especially useful in treating* pfmdr1* chloroquine-resistant isolates (IC_50_ = 14.3 nM) [51]. The docked conformer of amodiaquine in wtPfMDR1 and mtPfMDR1 showed that the functional residue Asn1042/Asp1042 which formed electrostatic interaction plays an important role in translocation to parasite cytoplasm. However, chloroquine bearing only one H-bond donor in its pharmacophore is not able to form H-bond both in wild and mutant types with the functional residues but makes extensive contacts with polar amino acids available in the drug-binding pocket and it can be one among the reasons for the development of chloroquine-resistant strains (IC_50_ = 245.1 nM) [51]. It can be noted that the binding energy of amodiaquine was found to be −7.21 kJ/mol, whereas chloroquine scored −4.93 kJ/mol. The variation in the binding energies of these 4-aminoquinoline drugs can be attributed to susceptibility and resistance of *P. falciparum* strains. 

Artemisinin, a sesquiterpene lactone containing trioxane pharmacophore or its derivatives, forms the standard treatment for most of the countries including India and is being prescribed in artemisinin combination therapy (ACT) along with long acting antimalarials including amodiaquine, lumefantrine, or mefloquine according to the recommendations of the National Programme of India, 2011 [2]. Artemisinin with its peroxide bridge efficiently formed H-bond and electrostatic contacts with key residues, Ser1034/Cys1034, in both wild and mutant types beside the polar amino acids interaction with hydrophobic groups. Artemisinin binding energy (−7.86 kJ/mol) and its biological activity (EC_50_ = 3.2 to 108 nM) [52] are comparable to those of amodiaquine and chloroquine and found to be biologically significant.

Halofantrine is a substituted phenanthrene drug and holds close resemblance to other antimalarials such as quinine and lumefantrine and is very effective (IC_50_ < 6 nM) against erythrocytic stages of all human Plasmodia species including the *P. falciparum* chloroquine-resistant strains [53]. The contacts of halofantrine were enhanced by its H-bonding ability with Asn1042/Asp1042 and *π* interaction with nearby aromatic residue, Phe1070, and possessed a binding energy of −5.7 kJ/mol. In addition, mtPfMDR1 interaction with halofantrine was found to be similar to wild form. Lumefantrine, another antimalarial of arylamino alcohol group, was found to be more efficient (IC_50_ = 90.1 nM) [54] in interacting with wtPfMDR1 and mtPfMDR1 utilizing its hydrophobic core and establishes *π* contact with the Phe1070 with no H-bond. However, the mode of interaction with wtPfMDR1 and mtPfMDR1 was strongly associated with tolerance/resistance to lumefantrine *in vitro *and its increased concentration known to restore its activity [54]. 

Mefloquine, an analogue of quinine, is an orally administered medication. A randomized trial of mefloquine and lumefantrine in ACT revealed that the former is well tolerated and prevented more new infections with respect to lumefantrine [55]. Mefloquine has a binding energy of −5.32 kJ/mol and made H-bonds with Asn1042 in wtPfMDR1, whereas the H-bonding ability was abolished in mtPfMDR1. Vinblastine, an alkaloid from *Vinca rosea*, was observed to be active in PfMDR1 transport in mutant form [5]. Its binding mode along with its similar active constituent, vincristine was studied. Vincristine, had a favorable binding energy (−8.28 kJ/mol) in contrast to vinblastine (−6.79 kJ/mol) owing to its extensive electrostatic interaction. An ethnobotanical approach and *in vitro* study identified an active ingredient called calotropegenin in the plant, *Calotropis procera, *possessing antimalarial activity against chloroquine-sensitive and chloroquine-resistant *P. falciparum* strains [56]. Calotropegenin is structurally similar to vinblastine and vincristine, considered to understand its binding mode which formed H-bonds and interacted electrostatically with the functional residue, Ser1034/Cys1034 (binding energy: −7.29 kJ/mol). The biological inhibitory activity of this molecule is being under current investigation in our laboratory. Thus, on relating to the biological activity, a strong association can be laid upon the complexities of the ligand dataset under study which can be further deciphered in the mode of interaction with functional amino acids, and the high ligand structural surface contributes to receptor binding. It is believed that enhanced contacts of ligand within the drug-binding pocket increase the binding affinity, whereas small hydrophobic molecules including chloroquine having no H-bond contacts make them easily eluted from the hydrophobic field prevailed inside TMDs which lead to drug resistance. Hence, it would be very useful to select phytochemicals and optimize their functional groups to enhance the intermolecular contacts, thereby increasing the binding affinity towards mtPfMDR1.

## 4. Conclusion

We reported here the development of PfMDR1 computational model systematically and evaluated its reliability using various structural and statistical measures. The stability of the PfMDR1 in inward-facing conformation was pursued by energy minimization approach and holds very close structural arrangement to *M. musculus* P-gp1. Structural neighbourliness relied on crystallographic protein data which was utilized to identify interfacial amino acids which play an important role in NBDs dimerization leading to outward-facing conformation. These molecular motions were studied using protein-protein docking and related to Eco-MsbA structure in its outward-facing conformation representing the mode of substrate transport. Further, the interaction pattern of selected antimalarials in wild-type and mutant-type PfMDR1 was investigated, which showed that the high ligand surface area as well as the electrostatic and hydrophobic contacts greatly improved the binding affinity beside the contacts made with functional amino acids. We also showed that phytochemicals with documented antimalarial activity have better interaction in comparison to long lasting antimalarials which demonstrated the need for selecting potent small molecules to experiment *in vitro*. 

## Supplementary Material

Supplementary Figure 1: Multiple sequence alignment of PfMDR1 with templates.
Supplementary Figure 2: Sequence profile and hydrophobicity plot of PfMDR1.
Supplementary Figure 3: Local model quality of *M. musculus* P-gp1 and PfMDR1.
Supplementary Figure 4: The proximity of EL1 and EL6 in PfMDR1 closed apo form.

## Figures and Tables

**Table 1:** List of amino acids predicted to be localized at the NBDs interface site.

Domains^a^	Amino acids interacting at the NBDs dimer site^b,c^
NBD I	388: Asp (0.70), 389: Thr (1.27), 390: Arg (0.94), 391: Lys (1.01), 392: Asp (0.69), 414: Glu (1.39), 415: Ser (1.28), 429: Leu (1.18), 462: Gln (0.73), 463: Asp (1.03), 465: Leu (1.08), 467: Phe (1.10), 468: Ser (0.92), 469: Asn (0.26), 555: Leu (0.18), 558: Ser (0.45), 559: Asn (0.49), 561: Ser (0.33), 562: Lys (0.39), 563: Leu (0.89), 564: Ser (0.71), 565: Gly (0.41), 567: Gln (0.08), 588: Glu (0.43), 591: Ser (0.84), 592: Ser (1.49), 593: Leu (1.03), 594: Asp (1.67), 595: Asn (1.77), 596: Lys (1.16), 599: Tyr (0.73), 621: His (0.65), 624: Ser (0.33).

NBD II	1256: Gln (0.03), 1257: Glu (0.43), 1297: Leu (0.96), 1298: Pro (1.35), 1299: Asn (0.93), 1300: Lys (0.05), 1301: Tyr (0.11), 1302: Asp (0.20), 1303: Thr (0.47), 1304: Asn (0.05), 1306: Gly (0.83), 1307: Pro (0.80), 1310: Lys (0.57), 1311: Ser (0.72), 1312: Leu (0.87), 1313: Ser (0.84), 1316: Gln (1.33), 1317: Lys (0.42), 1319: Arg (0.97), 1337: Glu (0.26), 1340: Ser (0.64), 1341: Ser (0.32), 1342: Leu (0.12), 1343: Asp (1.23), 1344: Ser (0.16), 1346: Ser (0.27).

^a^NBD: Nucleotide binding domain (I and II).

^b^Number preceding the amino acids indicate sequence position.

^c^Number within brackets indicate interfacial residue scores.

**Table 2:** Energetic details of ligand interaction with wild-type and mutant-type PfMDR1.

Compounds	Type of strain^a^	Amino acid interactions^b,c^
Amodiaquine	wtPfMDR1	H-bond: Tyr1046 Elec: **Asn1042**, Ser1043, Tyr1046 vdW: Phe74, Ile75, Val77, Phe78, Ile81, Ile94, Ser97, Leu98, Leu324, Trp1031, Ala1045, Phe1063, Met1064, Leu1067, Phe1070, Ile1071
	mtPfMDR1	H-bond: Tyr1046 Elec: **Asp1042**, Ser1043, Tyr1046 vdW: Ile81, Ser97, Leu324, Phe74, Val77, Phe78, Leu98, Ala1045, Phe1063, Leu1067

Artemisinin	wtPfMDR1	H-bond: **Ser1034**, Gln1035(2) Elec: Asn943, **Ser1034**, Gln1035, Gln1038 vdW: Ala1037
	mtPfMDR1	H-bond: **Cys1034**, Gln1035(2) Elec: Asn943, **Cys1034**, Gln1035, Gln1038 vdW: Phe947, Arg950, Trp1031, Ile1041, Phe1070

Calotropegenin	wtPfMDR1	H-bond: Asn943(2), **Ser1034**, Gln1038 Elec: Asn943, **Ser1034**, Gln1035, Ala1037, Gln1038 vdW: Phe947, Arg950, Trp1031, Ile1041, Phe1070
	mtPfMDR1	H-bond: Asn943, **Cys1034**, Gln1038(2), Elec: Asn943, **Cys1034**, Gln1035, Ala1037, Gln1038 vdW: Phe947, Arg950,Trp1031, Ile1041, Phe1070

Chloroquine	wtPfMDR1	H-bond: None Elec: **Asn1042** vdW: Phe74, Ile75, Phe78, Leu324, Ile328, Ile1041, Ala1045, Phe1063, Ser1066, Leu1067, Phe1070, Ile1071
	mtPfMDR1	H-bond: None Elec: Leu324, **Asp1042** vdW: Phe74, Ile75, Phe78, Ile328, Ile1041, Ala1045, Phe1063, Ser1066, Leu1067, Phe1070, Ile1071

Halofantrine	wtPfMDR1	H-bond: **Asn1042**(2) Elec: Gln1038, **Asn1042** Pi: Phe1070 vdW: Phe74, Leu71, Phe78, Tyr1046, Phe1063, Leu1067, Ile1071, Leu324
	mtPfMDR1	H-bond: **Asp1042** Elec: Gln1038, **Asp1042** vdW: Leu71, Phe74, Phe78, Leu324, Tyr1046, Phe1063, Leu1067, Phe1070, Ile1071

Lumefantrine	wtPfMDR1	H-bond: None Elec: Ser178, Met332, Asn339, Asn943, Gln1038 Pi: Phe174 vdW: Leu71, Ile171, Thr175, Ile335, Trp1031, **Ser1034**, Gln1035, Ala1037, Thr1073, Gly1074, Phe1070
	mtPfMDR1	H-bond: None Elec: Ser178, Met332, Asn339, Asn943, Gln1038 vdW: Leu71, Ile171, Phe174, Thr175, Ile335, Trp1031, **Cys1034**, Gln1035, Ala1037, Phe1070, Thr1073, Gly1074

Mefloquine	wtPfMDR1	H-bond: Gln1038, **Asn1042** Elec: Gln1038, **Asn1042** vdW: Leu71, Phe74, Phe78, Ala1045, Tyr1046, Phe1063, Leu1067, Phe1070, Ile1071.
	mtPfMDR1	H-bond: Gln1038 Elec: Gln1038, **Asp1042** vdW: Leu71, Phe74, Phe78, Ala1045, Tyr1046, Phe1063, Leu1067, Phe1070, Ile1071

Vinblastine	wtPfMDR1	H-bond: Gln1035(2) Elec: **Ser1034**, Gln1035, Gln1038, **Asn1042** vdW: Leu71, Phe74, Ile75, Leu324, Leu327, Ile328, Phe331, Met332, Ile335, Asn943, Leu1067, Phe1070, Ile1071, Gly1074
	mtPfMDR1	H-bond: Gln1035(2) Elec: **Cys1034**, Gln1035, Gln1038, **Asp1042** vdW: Leu71, Phe74, Ile75, Leu324, Leu327, Ile328, Phe331, Met332, Ile335, Asn943, Leu1067, Phe1070, Ile1071, Gly1074

Vincristine	wtPfMDR1	H-bond: None Elec: Asn943, Gln1038, Phe1070, Ile1071, Gly1074, Ser1075 vdW: Leu71, Ile75, Ile328, Phe947, Arg950, **Ser1034**, Gln1035, Ala1037, **Asn1042**, Ala1077
	mtPfMDR1	H-bond: None Elec: Asn943, Gln1038, Phe1070, Ile1071, Gly1074, Ser1075 vdW: Leu71, Ile75, Ile328, Phe947, Arg950, **Cys1034**, Gln1035, Ala1037, **Asp1042**, Ala1077

^a^wtPfMDR1: wild-type PfMDR1.

^a^mtPfMDR1: mutant-type PfMDR1.

^b^H-bond: Hydrogen bond.

^b^Elec: Electrostatic interaction.

^b^vdW: van der Waals interaction.

^c^Function residues are depicted in bold face.

^c^Number within brackets indicate the number of H-bonds formed.
