# Supplementary material for: A Computational Approach towards the Understanding of Plasmodium falciparum Multidrug Resistance Protein 1
Source: ISRN Bioinform. 2013 Aug 1;2013:437168. doi: 10.1155/2013/437168 (PMC4393060; doi:10.1155/2013/437168)
Supplement: Supplementary file 1 — Supplementary Figure 1: Multiple sequence alignment of PfMDR1 with templates. Supplementary Figure 2: Sequence profile and hydrophobicity plot of PfMDR1. Supplementary Figure 3: Local model quality of M. musculus P-gp1 and PfMDR1. Supplementary Figure 4: The proximity of EL1 and EL6 in PfMDR1 closed apo form. [file 437168.f1.pdf]

## Electronic Supplementary files

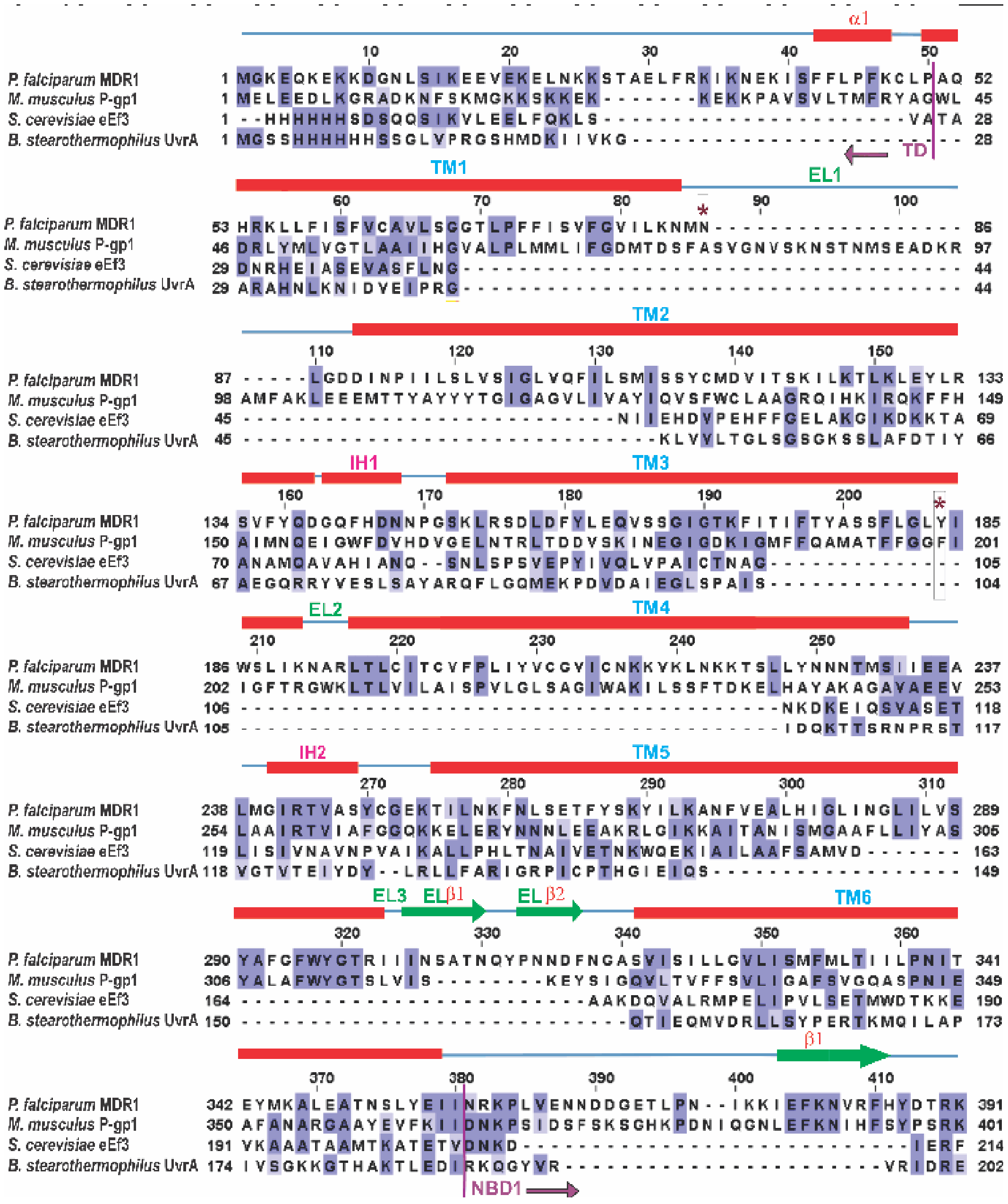

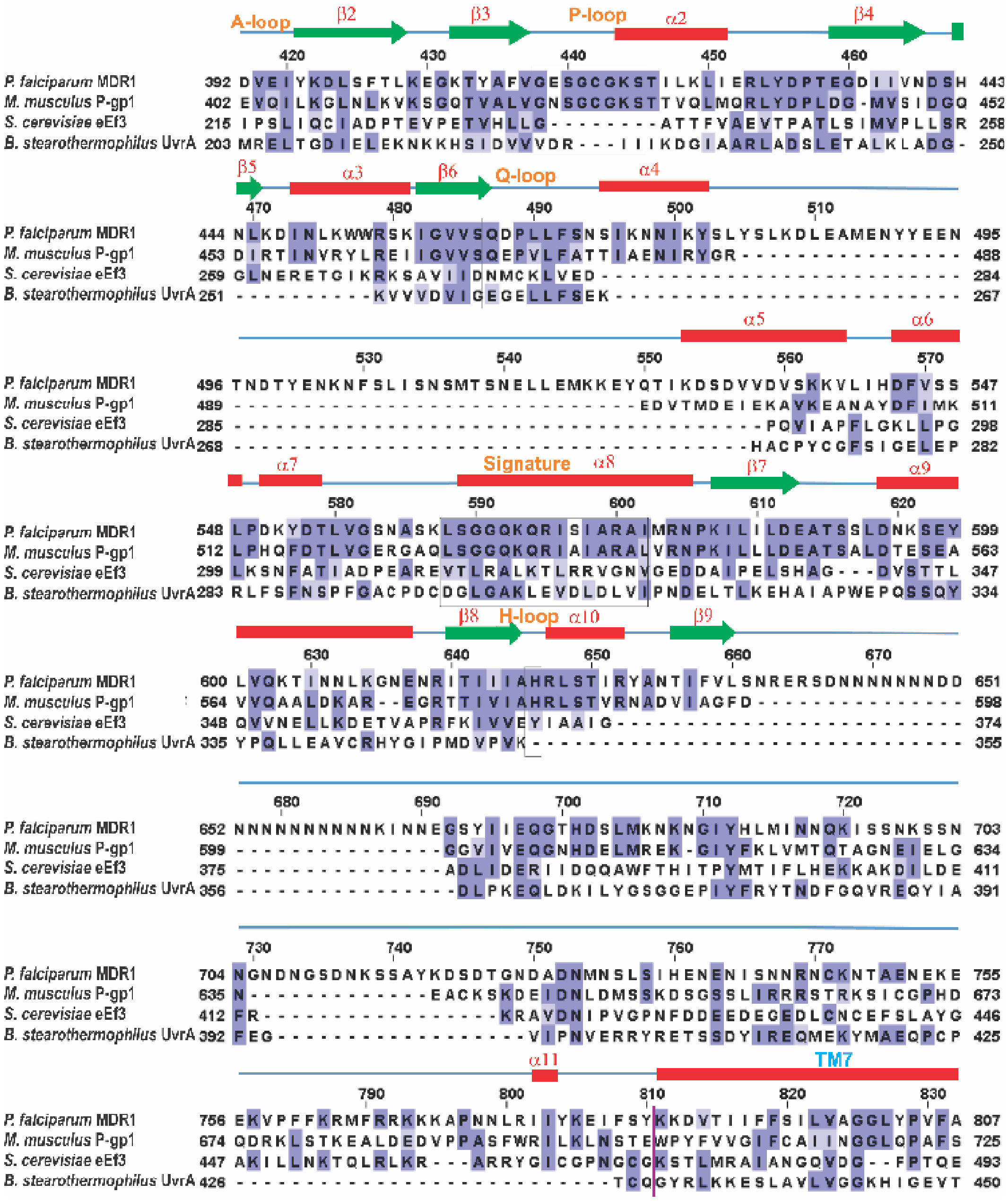
A-loop
β2
β3
P-loop
α2
β4
P. falciparum MDR1
M. musculus P-gp1
S. cerevisiae eEf3
B. stearothermophilus UvrA
β5
α3
β6
Q-loop
α4
α5
α6
α7
Signature
α8
β7
α9
β8
H-loop
α10
β9
α11
TM7

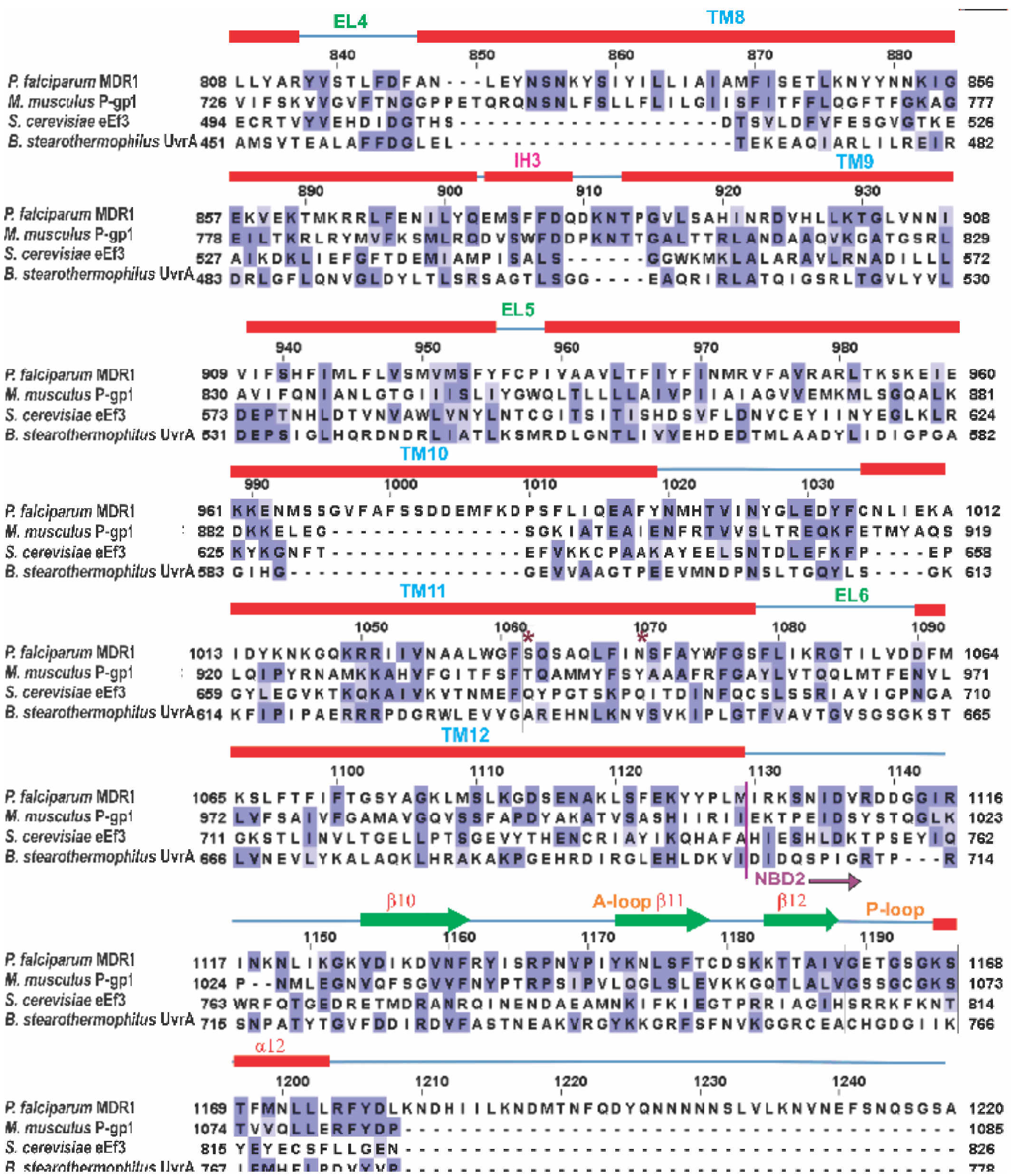
EL4
TM8
IH3
TM9
EL5
TM10
TM11
EL6
TM12
NBD2
β10
A-loop β11
β12
P-loop
α12
P. falciparum MDR1
M. musculus P-gp1
S. cerevisiae eEf3
B. stearothermophilus UvrA

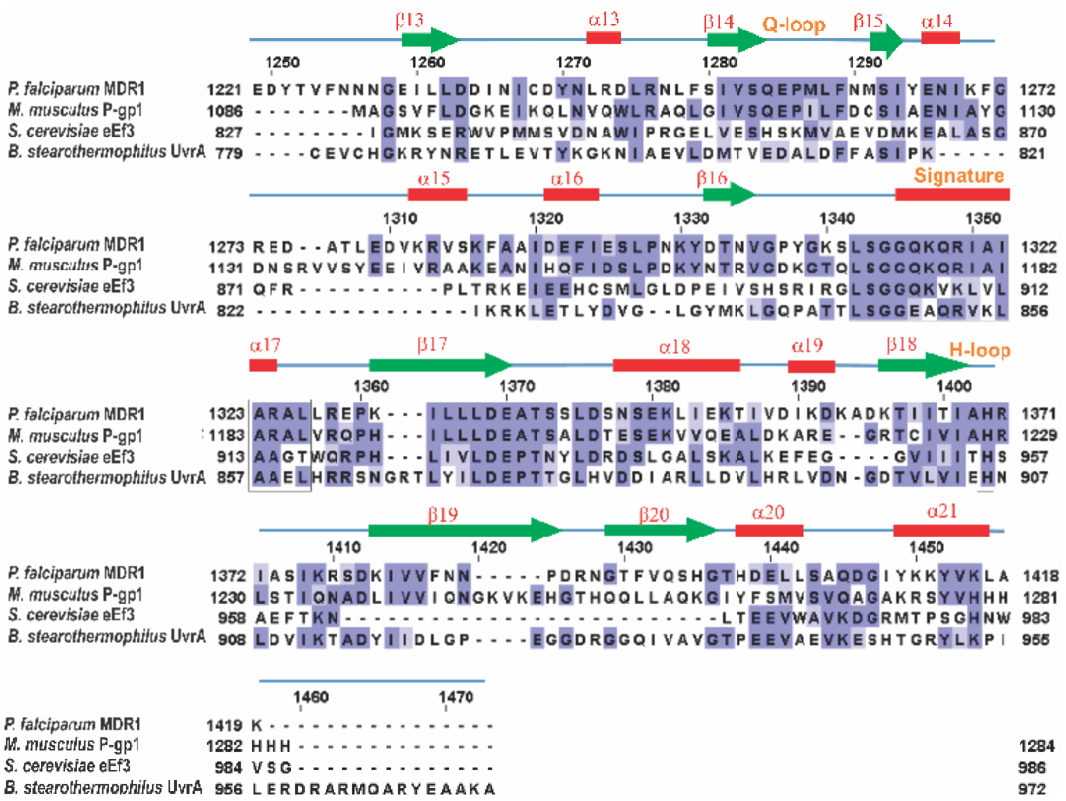

**Suppl. Fig. 1** Multiple sequence alignment of PfMDR1 with templates

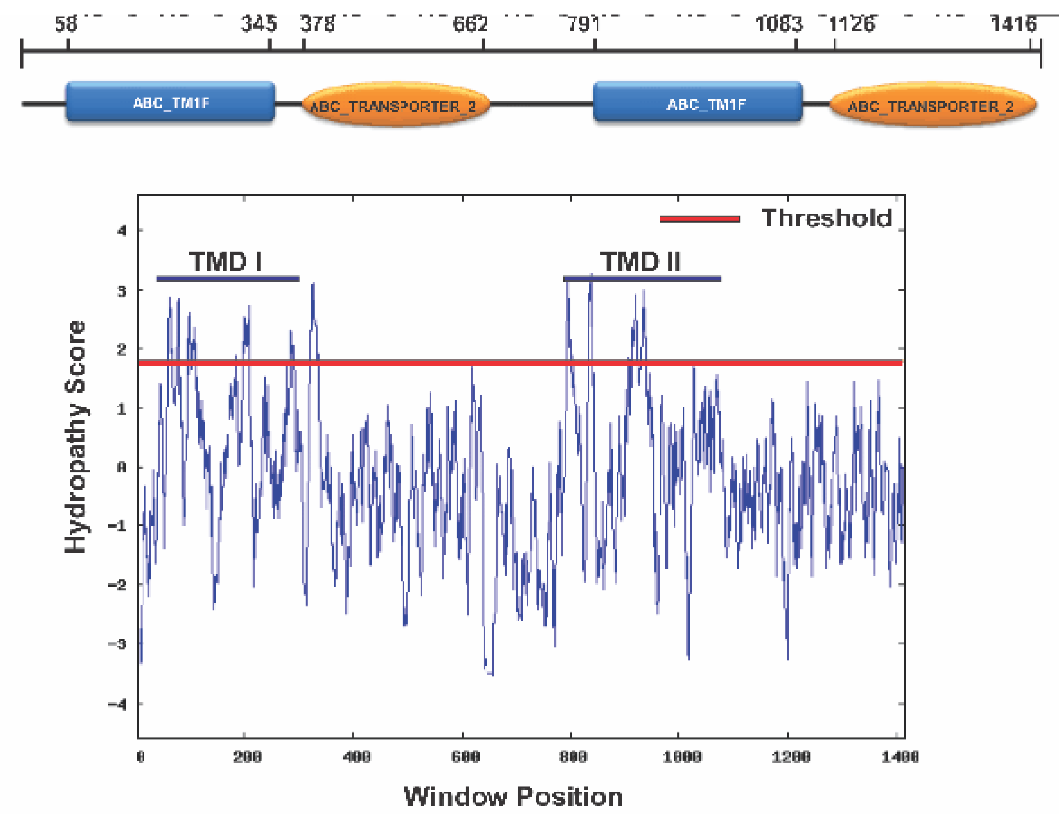


**Suppl. Fig. 2** Sequence profile and hydrophobicity plot of PfMDR1

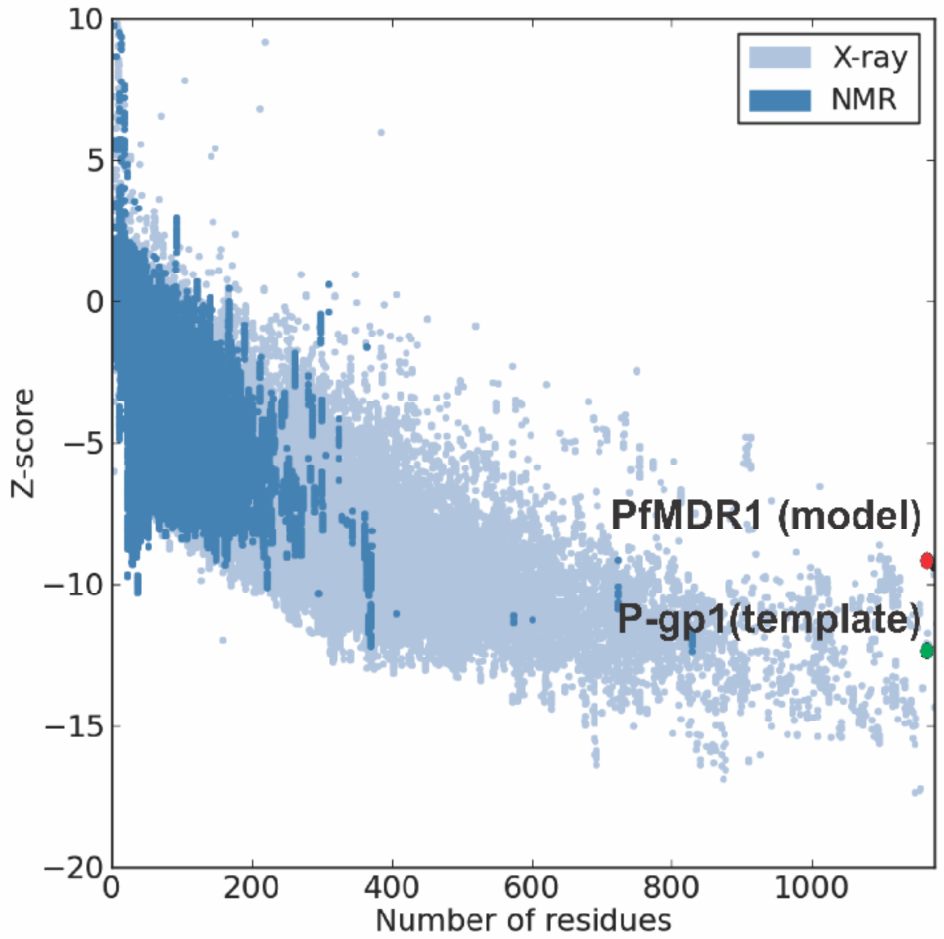


**Suppl. Fig. 3** Local model quality of *M. musculus* P-gp1 (green dot) and PfMDR1 (red dot)

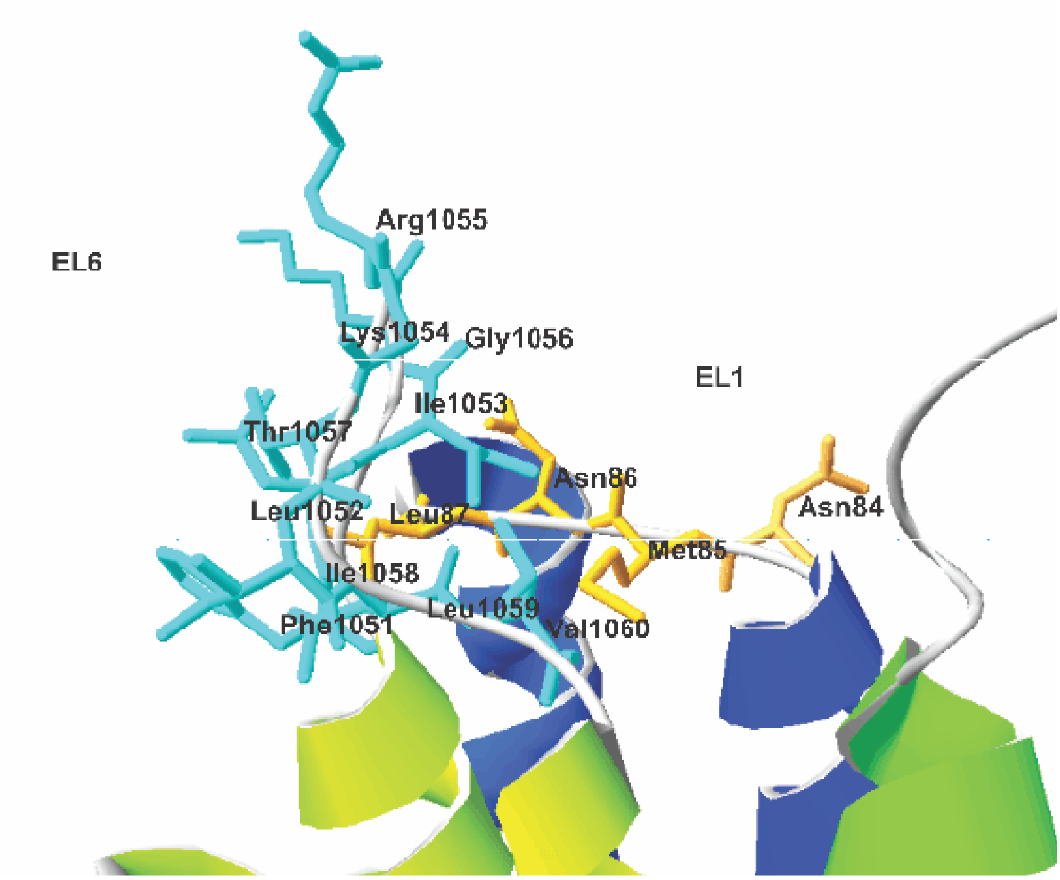


**Suppl. Fig. 4** The proximity of EL1 and EL6 in PfMDR1 closed apo form
